# Remdesivir for the Treatment of COVID-19: A Systematic Review of the Literature

**DOI:** 10.5811/westjem.2020.5.47658

**Published:** 2020-05-20

**Authors:** Arif Musa, Kasim Pendi, Areio Hashemi, Elizabeth Warbasse, Sarkis Kouyoumjian, Jenna Yousif, Emily Blodget, Susan Stevens, Besma Aly, David A. Baron

**Affiliations:** * Wayne State University School of Medicine, Detroit, Michigan; † Musa Biomedical Consulting, Anaheim, California; ‡ Southern California University of Health Sciences, School of Professional Studies, Whittier, California; § William Carey University, College of Osteopathic Medicine, Hattiesburg, Mississippi; ¶ Wayne State University School of Medicine, Department of Emergency Medicine, Detroit, Michigan; || University of Southern California Keck School of Medicine, Division of Infectious Diseases, Department of Medicine, Los Angeles, California; # Western University of Health Sciences, Office of the Provost, Pomona, California

## Abstract

In March 2020, the World Health Organization declared the spread of SARS-CoV-2 a global pandemic. To date, coronavirus disease-2019 (COVID-19) has spread to over 200 countries, leading to over 1.6 million cases and over 99,000 deaths. Given that there is neither a vaccine nor proven treatment for COVID-19, there is currently an urgent need for effective pharmacotherapy. To address the need for an effective treatment of SARS-CoV-2 during the worldwide pandemic, this systematic review of intravenous (IV) remdesivir was performed. Remdesivir, an anti-viral prodrug originally developed to treat Ebola virus disease, has shown broad spectrum activity against the Coronavirus family. A recent case report reported improvement of clinical symptoms with remdesivir in a patient with COVID-19. After conducting a systematic search of 18 clinical trial registries and three large scientific databases, we identified 86 potentially eligible items. Following removal of duplicates (n = 21), eligible studies were reviewed independently by two authors. After the first round of screening, inter-rater agreement was 98.5% (κ = 0.925). After the second round of full-text screening, inter-rater agreement was 100%. A total of seven ongoing and recruiting clinical trials of remdesivir (100–200 milligrams, intravenous [IV]) were included. We identified the following primary outcomes: patients discharged (n = 2); time to clinical status improvement (n = 2); improved O2 saturation (n = 2); body temperature normalization (n = 2); and clinical status (n = 1). Secondary outcomes in all identified studies included documentation of adverse events. Phase 3 trials are expected to be completed between April 2020–2023. Therefore, despite supportive data from in vitro and in vivo studies, the clinical effectiveness of IV remdesivir for treatment of COVID-19 and potential side effects remain incompletely defined in the human population.

## INTRODUCTION

The novel coronavirus outbreak, which began as an epidemic in Wuhan, China, in December 2019 has been confirmed to share 79.6% sequence identity with SARS-CoV and 96% genome identity with a coronavirus species in bats, its natural reservoir.^1^ Initially referred to as 2019-nCoV, the virus has been renamed SARS-CoV-2, and the disease that results is coronavirus disease-2019 (COVID-19).^2^ At the time of this authorship, there are over 1.6 million confirmed cases and over 99,000 deaths in 205 countries worldwide.^3^ On March 11, 2020, the World Health Organization responded to the unprecedented spread of COVID-19 and inaction of international governments by declaring the outbreak a pandemic.^4^

There is currently no safe and proven treatment for COVID-19 and there is no vaccine for SARS-CoV-2; however, vaccines are under development and several treatments have been proposed and are under investigation.^5^–^6^ The rapid international spread and severity of COVID-19, which causes symptoms varying from fever, dry cough, and shortness of breath to diarrhea and body aches, has spurred the greater scientific community to quickly identify treatments for the disease.^4^ Potential pharmacological treatments for COVID-19 may be found in one of three categories: broad-spectrum anti-viral drugs; repurposed existing drugs or substances; and novel therapeutic agents.^7^ We chose to analyze remdesivir based on established inhibition of infection by the novel coronavirus in human cell lines (human liver cancer HuH-7 cells).^8^

Remdesivir falls into the first category as an anti-viral prodrug developed to treat infections caused by viruses of the family *Filoviridae*, which includes Ebola virus (*Zaire embolavirus*).^9^ Discovered in 2016 small molecule GS-5734, remdesivir was used initially to treat Ebola virus disease (EVD) as an adenosine analog that incorporates into viral RNA, leading to premature chain termination and inhibition of viral replication.^10^ But in 2019, the first confirmed case of COVID-19 in the United States prompted the use of intravenous (IV) remdesivir for compassionate use, leading to marked improvement of the patient’s clinical status within 24 hours.^11^ The authors suggested that additional clinical studies were needed to complete the safety and efficacy profiles of the anti-viral drug. Given the worldwide urgency for an effective and safe treatment for COVID-19 and the therapeutic potential of remdesivir, this systematic review was performed to determine the outcomes and adverse events associated with this investigational, anti-viral medication.

## METHODS

We performed a systematic review of the use of remdesivir for treatment of COVID-19. Eligibile articles included human patients with SARS-CoV-2 infection, remdesivir administration, patient outcomes, and adverse events. A search strategy was developed for each database without restrictions for language or years considered. The search parameters for Embase were as follows:

1. remdesivir OR GS-5734
2. coronavirus OR coronaviruses OR 2019-nCoV OR COVID-19 OR SARS-CoV-2 OR SARS-COV2
3. #1 AND #2

Clinical trial registries that were searched included the following: clinicaltrials.gov; Chinese Clinical Trial Registry (ChiCTR); Australian New Zealand Trial Registry; Brazilian Clinical Trials Registry; Chinese Research Information Service Republic of Korea; Clinical Trials Registry India; Cuban Public Registry of Clinical Trials; German Clinical Trials Register; Iranian Registry of Clinical Trials; International Standard Randomised Controlled Trials Number Registry; Japan Primary Registries Network; Lebanese Clinical Trials Registry; Thai Clinical Trials Registry; The Netherlands National Trial Register; Pan African Clinical Trial Registry; Peruvian Clinical Trial Registry; and Sri Lanka Clinical Trials Registry. The following databases were searched: Embase, PubMed, and Web of Science. The search was last updated on March 17, 2020. Study coordinators were contacted for additional information if appropriate.

Eligible studies were identified and screened according to inclusion-exclusion criteria that were established a priori (Table 1). Two authors independently screened the search results, and a third author resolved the disputes. Inter-rater agreement was quantified by Cohen’s kappa scores as well as percentage agreement as recommended by McHugh.^12^ Studies were first screened by title and abstract and then by full-text review. Data were extracted by one author and included the following items: type of study; intervention; number of participants; patient outcomes; adverse events; and study characteristics. We used the Preferred Reporting Items for Systematic Reviews and Meta-Analyses (PRISMA) statement as an aid in this report.^13^ There is no online review protocol for this study.

## RESULTS

The database search yielded a total of 86 items from the following databases: Embase (*n* = 21), PubMed (*n* = 20), Web of Science (*n* = 28), European Union Clinical Trials Register (*n* = 2), and clinicaltrials.gov (*n* = 8). After removal of duplicates (*n* = 21), the first round of screening yielded eight potentially eligible items. Studies were excluded for meeting any of the following criteria: non-human study (*n* = 3); review or meta-analysis (*n* = 15); not including SARS-CoV-2 (*n* = 16); or multiple criteria met (*n* = 23). Inter-rater agreement following the first round of screening was 98.5% (κ = 0.925). After the second round of screening, seven items were included. Inter-rater agreement was 100%. Study characteristics are described in Table 2.

This review of remdesivir identified ongoing and recruiting trials in 11 countries, including the United States, China, Taiwan, France, and Italy. The average number of participants was 450 (range = 308–600). Selection criteria for each trial varied according to the severity of symptoms (based on peripheral capillary oxygen saturation). Five trials involved a 200-milligram (mg) intravenous (IV) loading dose following by maintenance dose of 100 mg for nine days. Two trials involved a single, 100-mg IV infusion. The primary outcomes for each trial were as follows: proportion of patients discharged (*n* = 2); time to clinical improvement (*n* = 2); improved oxygen saturation (*n* = 2); normalization of body temperature (*n* = 2); and percentage of each severity rating on a 7-point ordinal scale to assess clinical status (*n* = 1). Secondary outcomes included adverse events (*n* = 7); length of stay (*n* = 2); mortality (*n* = 3); duration of ventilation or supplemental oxygen use (*n* = 3); and reduction in viral load (*n* = 2). Results are expected in April 2020 (*n* = 2), May 2020 (*n* = 2), and April 2023 (*n* = 1).

## DISCUSSION

Our systematic search identified a total of 86 studies eligible for inclusion, of which seven were incorporated into a qualitative synthesis. All seven of the included studies were Phase 3 clinical trials that were either recruiting patients or considered ongoing. In each trial, IV remdesivir (100–200 mg) was the primary intervention. The predominant treatment protocol described a 200 mg dose administered on the first day followed by subsequent doses of 100 mg each following day (for a total of 5 or 10 days depending on the treatment arm). However, none of the included studies have reported completed or partial data. As a result, the clinical utility of remdesivir for the treatment of COVID-19 remains to be seen, and any adverse events have yet to be reported.

As early as 2017, Sheahan et al reported GS-5734 activity against MERS-CoV and SARS-CoV in human lung cells, suggesting that remdesivir may prove effective against endemic and emerging coronaviruses.^14^ Agostini et al later reported that GS-5734 effectively inhibited coronavirus replication in vivo despite intact exoribonuclease proofreading, indicating that remdesivir may have utility against resistant coronavirus strains.^15^ Further research has demonstrated the broad-spectrum activity of remdesivir for the purpose of treating endemic coronavirus infections.^16^

In December 2019, the first case of SARS-CoV-2 in the United States was successfully treated by IV remdesivir without adverse effects.^11^ The patient was treated with remdesivir on hospital day 7, and on day 8 the patient experienced symptomatic and clinical improvement significant enough to discontinue supplemental oxygenation with improved saturation in room air, as well as resolution of rales and anorexia. Remdesivir was first reported for treatment of EVD in 2016 with subsequent studies indicating mixed results.^10^, ^17^–^18^ Ko et al argued that the findings from previous studies of remdesivir for EVD support testing of remdesivir for treatment of COVID-19.^19^ Concurrent in vitro research has supported the use of the drug to treat SARS-CoV-2 infections.^20^ In contrast, Zhang et al have raised concerns about the possibility of unknown adverse reactions.^8^

## LIMITATIONS

The primary limitation of this systematic review stems from the lack of reported patient outcomes from human trials, which are in varying phases of completion. Although some clinical trial registries display preliminary reports of ongoing trials, these partial data are not available for quantitative analysis. Trials are scheduled to be completed as early as April–May 2020.

## CONCLUSION

There is both in vitro and limited clinical evidence that supports the use of remdesivir to treat SARS-CoV-2. However, Phase 3 clinical trials have not yet been completed and partial data has not yet been reported. The side-effects profile of remdesivir remains similarly not well defined. Until high-quality studies report significant improvements with administration of IV remdesivir, the use of this experimental drug should be limited to randomized controlled trials. Therefore, the potential of remdesivir as a standard of care therapy for COVID-19 remains to be determined.

**NOTE:** An addenum to this article has been written by the author AM.^20^

## Figures and Tables

**Table 1:** Inclusion-exclusion criteria for systematic review of studies regarding the use of remdesivir for treatment of COVID-19.

Inclusion	Exclusion
1. Human study	1. In vitro study or In vivo study (e.g. animal model) or Other non-human study (unless can be isolated)
2. Remdesivir (GS-5734) included	2. Remdesivir (GS-5734) not included in the article
3. Case report or Case series or Letter of correspondence or Observational study or Clinical trial or Randomized controlled trial	3. Literature review or Systematic review or Meta-analysis or
4. Novel coronavirus or 2019-nCov or SARS-COV-2 or COVID-19	4. Other pathogen or virus included (e.g. Marburg)

Studies that meet all the inclusion criteria may be included in the systematic review. Studies that meet any of the exclusion criteria should be excluded from the systematic review.

**Table 2:** Study characteristics regarding the use of remdesivir for treatment of COVID-19.

ID	Trial Status	Country	Number of Sites	Phase of Trial	Intervention	Number of Participants	Primary Outcome(s)
NCT04292899	Recruiting	Hong Kong Republic of Korea Singapore United States	10	3	Intravenous RDV 200 mg on Day 1 followed by Intravenous RDV 100 mg for 4 days Intravenous RDV 200 mg on Day 1 followed by Intravenous RDV 100 mg for 9 days	400	Improved oxygen saturation; Normalization of body temperature
NCT04292730	Recruiting	Hong Kong Republic of Korea Singapore United States	10	3	Intravenous RDV 200 mg on Day 1 followed by Intravenous RDV 100 mg for 4 days Intravenous RDV 200 mg on Day 1 followed by Intravenous RDV 100 mg for 9 days	600	Proportion of discharged patients
NCT04257656	Recruiting	China	1	3	Intravenous RDV 200 mg on Day 1 followed by Intravenous RDV 100 mg for 9 days	453	Time to clinical improvement (restricted to 28 days)
NCT04252664	Recruiting	China	1	3	Intravenous RDV 200 mg on Day 1 followed by Intravenous RDV 100 mg for 9 days	308	Time to clinical improvement (restricted to 28 days)
NCT04280705	Recruiting	Republic of Korea Singapore United States	20	3	Intravenous RDV 200 mg on Day 1 followed by Intravenous RDV 100 mg for 9 days	394	Percentage of each severity rating on 7-point ordinal scale with a 15 day time frame
2020-000841-15	Ongoing*	China France Germany Hong Kong Italy Japan Republic of Korea Singapore Spain Taiwan United States	15	3	Intravenous RDV 100 mg	400	Improved oxygen saturation; Normalization of body temperature (restricted to 14 days)
2020-000842-32	Ongoing*	China France Germany Hong Kong Italy Japan Republic of Korea Singapore Spain Taiwan United States	15	3	Intravenous RDV 100 mg	600	Proportion of discharged participants (restricted to 14 days)

* Trial status (as defined by ClinicalTrials.gov) regards a trial as ‘ongoing’ if it had one of these statuses: ‘Active, not recruiting’, ‘Available’, ‘Enrolling by invitation’, ‘Not yet recruiting’, ‘Recruiting’, or ‘Suspended’.

*RDV*, Remdesivir; *mg*, milligrams.
